# The Implementation of Improvement Interventions for "Low Performing" and "High Performing" Organisations in Health, Education and Local Government: A Phased Literature Review

**DOI:** 10.34172/ijhpm.2020.197

**Published:** 2020-11-01

**Authors:** Cecilia Vindrola-Padros, Jean Ledger, Estela Capelas Barbosa, Naomi J. Fulop

**Affiliations:** ^1^Department of Applied Health Research, University College London, London, UK.; ^2^Bristol Medical School, University of Bristol, Bristol, UK.

**Keywords:** Low-Performing, High-Performing, Improvement, Interventions, Healthcare Organisations

## Abstract

**Background:** There is limited understanding about whether and how improvement interventions are effective in supporting failing healthcare organisations and improving the quality of care in high-performing organisations. The aim of this review was to examine the underlying concepts guiding the design of interventions aimed at low and high performing healthcare organisations, processes of implementation, unintended consequences, and their impact on costs and quality of care. The review includes articles in the healthcare sector and other sectors such as education and local government.

**Methods:** We carried out a phased rapid systematic review of the literature. Phase one was used to develop a theoretical framework of organisational failure and turnaround, and the types of interventions implemented to improve quality. The framework was used to inform phase 2, which was targeted and focused on organisational failure and turnaround in healthcare, education and local government settings. We used the Preferred Reporting Items for Systematic Reviews and Meta-Analyses (PRISMA) statement to guide the reporting of the methods and findings and the Mixed Methods Appraisal Tool (MMAT) as a quality assessment tool. The review protocol was registered with PROSPERO (CRD: 42019131024).

**Results:** Failure is frequently defined as the inability of organisations to meet pre-established performance standards and turnaround as a linear process. Improvement interventions are designed accordingly and are focused on the organisation, with limited system-level thinking. Successful interventions included restructuring senior leadership teams, inspections, and organisational restructuring by external organisations. Limited attention was paid to the potential negative consequences of the interventions and their costs.

**Conclusion:** Dominant definitions of success/failure and turnaround have led to the reduced scope of improvement interventions, the linear perception of turnaround, and lack of consideration of organisations within the wider system in which they operate. Future areas of research include an analysis of the costs of delivering these interventions in relation to their impact on quality of care.

## Introduction

 There may be indications of persistent performance or quality issues in a healthcare organisation long before a crisis comes to the attention of the wider public and regulators. This highlights the need for transparent, integrated, and timely processes for identifying quality and safety issues within organisations and across healthcare systems.^1^ Attention has been placed on failing healthcare organisations, their characteristics and the factors (both internal and external) that might lead to low performance. These include for example low leadership capability, (as indicated by, eg, lack of ability to engage with staff, or to be transparent), ‘closed’ culture, and antagonistic external relationships.^2,3^ There are also a number of analyses of organisational failure and sometimes turnaround in the business sector, some of these including high profile corporate failures, such as Enron, Marks and Spencer^4^ and the financial crash of 2008,^5^ which identify reasons for failure and how they might be addressed.

 A recent systematic review of research on the characteristics of failing healthcare organisations in multiple countries and settings identified five characteristics shared across failing organisations: (1) poor organisational culture; (2) inadequate infrastructure; (3) lack of a cohesive mission; (4) system shocks; and (5) dysfunctional external relations with other hospitals, stakeholders or governing bodies.^6^ More specifically, a hierarchical culture and leadership focused on avoiding penalties and achieving financial targets – rather than a patient-centred mission – are characteristics identified in many failing healthcare organisations.^5,7,8^ High-performing organisations share features such as organisational cultures that embrace change, actively engage members of staff in decision-making processes and improvement interventions and seek to build partnerships with other organisations to share learning and good practice.^6^

 Available reviews, such as that by Vaughn et al,^6^ suggest that an important next step after diagnosis of problems is the development of high-quality interventions capable of helping struggling healthcare organisations to improve. However, there is limited understanding about whether and how improvement interventions are effective in supporting failing organisations and improving the quality of care in high-performing organisations in the public sector. The aim of this review is to examine the underlying concepts guiding the design of these interventions, processes of implementation and unintended consequences of implementing the interventions, and their impact on costs and quality of care. The review includes articles in the healthcare sector as well as other public sectors such as education and local government, to learn from the extensive research carried out in these non-healthcare sectors.

## Methods

###  Design

 The review was based on the phased rapid review method proposed by Tricco et al^9^ and expanded the review of organisational failure published by Vaughn et al.^6^ The rapid review method followed a systematic review approach, proposing adaptations to some of the steps to reduce the amount of time required to carry out the review (ie, the use of large teams to review abstracts and full texts, and extract data; in lieu of dual screening and selection, a percentage of excluded articles is reviewed by a second reviewer, and software is used for data extraction and synthesis, as appropriate^9^).

 The review included two phases. Phase one was based on a broad search of health services, business and management journals, and a review of the grey literature (eg, think tank reports) to develop a theoretical understanding of the main characteristics of organisational failure and turnaround, success and the types of interventions implemented to improve quality (for an example of this approach see Ferlie et al^10^). This literature was used to develop a conceptual and theoretically informed framework (see Table 1 in results section). The framework was used to inform the phase 2 research questions, search strategy, inclusion criteria and interpretation of findings.

**Table 1:** Main Characteristics of the Included Studies

	**Education**	**Local Government**	**Healthcare**	**Total**
Number of studies	18	9	15	42
Study location	8 UK 9 USA 1 European comparison	8 UK 1 Israel	4 UK 8 USA 1 UK and USA 1 Israel 1 Canada	20 UK 17 USA 1 UK and USA 1 Canada 2 Israel 1 European comparison
Publication date range	1999-2019	2004-2014	2005-2018 (most 2010 onward)	1999-2019 (most post 2010)
Study design	12 Qualitative 3 Mixed-methods 3 Quantitative	4 Qualitative 3 Mixed-methods 2 Quantitative	9 Qualitative 6 Quantitative	25 Qualitative 6 Mixed-methods 11 Quantitative

 We used the Preferred Reporting Items for Systematic Reviews and Meta-Analysis (PRISMA) statement^11^ to guide the reporting of the methods and findings. The review protocol was registered with PROSPERO (CRD: 42019131024).

###  Research Questions

 The review sought to answer the following questions:

*Phase 1 (covering health services research, management and business studies) *

1. How are ‘failing organisations’ defined?
2. What are the theoretical approaches that have been used to explain organisational failure?
3. How is ‘organisational turnaround’ defined?
4. Which theoretical approaches have been used to study turnaround strategies (if any)?

*Phase 2 (covering healthcare, education and local government) *

5. What are the main interventions used to improve quality?
6. Do the studies highlight any specific issues with implementation?
7. What are the interventions classified as ‘successful’?
8. Have any of these interventions been evaluated? If so, what is the impact and sustainability of improvements produced by these interventions?
9. What are the costs of these interventions?

###  Phase 1

**Figure 1:**
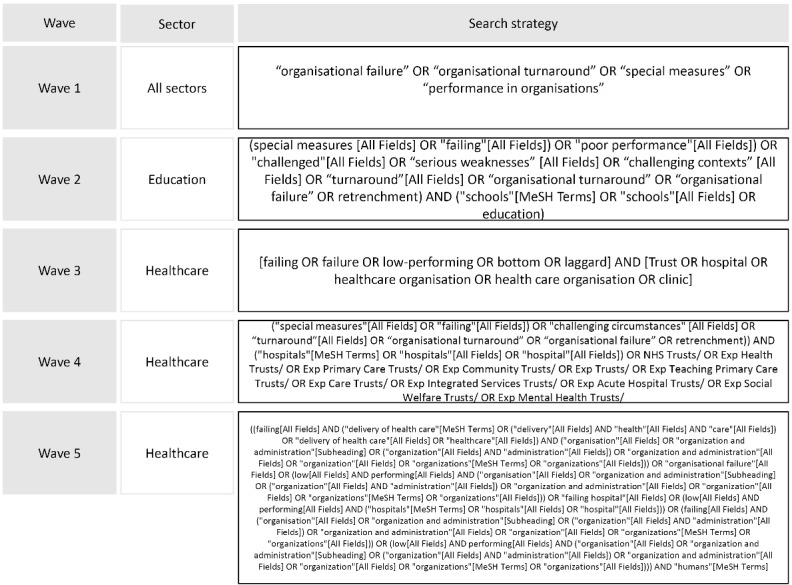
Snowball Technique for Five Waves of Searches (Phase 1).

 We used a phased search approach.^9^ The first phase was broad, covering literature from the fields of health services research, management and business studies to identify overarching themes and definitions on regulation, performance and quality improvement in healthcare organisations and the public sector. Broad terms such as “organisational failure,” “organisational turnaround,” “special measures” and “performance in organisations” were used to identify initial relevant literature across the public sector. A second search targeted literature in the education sector. All other searches (3-5) focused on the health sector. Using a snowball technique, additional terms were found and inserted into a search strategy for five databases (MEDLINE, EMBASE, CINAHL Plus, Web of Science, and Open Grey), creating longer and more complex search strategies (see Figure 1). These databases were selected in consultation with a librarian who sought to identify the most relevant ones for the review topic.

 Phase 1 focused on identifying the theoretical content from the literature on organisational failure and turnaround to develop a thematic framework to guide the review. We followed the approach for building thematic frameworks for reviews used by Ferlie et al.^10^ Definitions for key concepts such as “organisational failure/success” and “turnaround” were identified. Furthermore, we searched for the main theoretical frameworks used to explain these processes and synthesized their main characteristics. We sought to create a high-level overview of the different perspectives that have been used to explore failure, success and turnaround in organisations. The findings from phase 1 informed the research questions developed to guide phase 2 of the review.

###  Phase 2 

####  Search Strategy

 The second phase was more targeted and focused only on organisational failure, success and turnaround in healthcare, education and local government settings. The search strategy (see Supplementary file 1) was designed in relation to the PICOS framework, the findings from phase one and strategies used in other reviews on improvement and low and high-performing organisations.^6,12,13^ We conducted a review of published literature using multiple databases: MEDLINE, CINAHL PLUS, EMBASE, and Web of Science. We searched for relevant grey literature using Open Grey and TRIP. Results were combined into Mendeley and duplicates removed. The reference lists of included articles (including grey literature) were screened to identify additional relevant publications. We also hand searched other relevant databases such as the King’s Fund library.

###  Selection

 Following rapid review methodology,^9^ one researcher screened the articles (including those published in peer-reviewed journals and grey literature) in the title phase, and three researchers cross-checked 20% of exclusions in the abstract and full-text phases. Disagreements were discussed until consensus was reached. The inclusion criteria used for study selection was: (1) focus on the delivery of interventions in failing organisations, defined as not meeting the required quality standards (self-defined), (2) focus on the delivery of interventions in high-performing organisations (self-defined), (3) describes empirical research, (4) describes a study in a healthcare, education or local government setting, (5) published in last 20 years, and (6) published in English.

###  Data Extraction and Management

 The included articles were analysed using a data extraction form developed in REDCap (Research Electronic Data Capture). The form was developed after the initial screening of full-text articles and piloted independently by two researchers using a random sample of five articles. Disagreements were discussed until consensus was reached. The data extraction form was finalised based on the findings from the pilot.

###  Data Synthesis

 Data were exported from REDCap and the main article characteristics were synthesised. The information entered in free text boxes was exported from REDCap and analysed using framework analysis.^14^ We used the thematic framework developed in the first stage of the review to guide our exploration of themes.

###  Quality Assessment

 We used the Mixed Methods Appraisal Tool (MMAT) to assess the quality of the included articles.^15^ Two researchers rated these articles independently. In cases of disagreement, the raters discussed their responses until consensus was reached. Inter-rater reliability was calculated using the kappa statistic.^16^ The quality assessment was not used as inclusion/exclusion criteria, only to understand the quality of the reviewed studies.

## Results

###  Phase 1 Thematic Framework

 The five waves of searches for phase 1 provided a working list of 56 relevant publications. The main components of this framework and key examples of this literature are included in Supplementary file 2 as reference. We found that four definitions of failure are common in the literature (as decline, crisis, performance reaching below a previously established performance level, and performance impeded by structural factors). However, only the latter considers failures at a system level (ie, beyond individual organisations and including multiple organisations). Some authors argued that failure and success should not be considered discrete, opposite concepts, but should be understood as in a dialectical relationship (highlighting the contradictions and inherent tensions between components). Five of the theoretical frameworks used to explain organisational success or failure (industrial organization, organisational ecology theory, life cycle theory, organisational psychology, failure and organisational learning/organisational culture, role of emotions) reproduce this focus on the organisation as the unit of analysis and neglect of system-level pressures, with the exception of two of the more recent ones (Failure and success within regimes of surveillance and Contextual factors leading to failure). Concepts of turnaround tend to privilege a linear conceptualisation of organisational recovery processes, with only one approach considering turnaround as a non-linear complex process. Turnaround has also been explored as an internal vs. external approach, with limited discussion of the interaction between internal and external strategies.

 The findings from phase 1 informed the research questions developed to guide phase 2 of the review. We sought to explore the interventions delivered in low-performing and high-performing organisations, identifying the underlying ideas that guided them such as their conceptualisation of failure/success as an organisational or system-wide feature, the perception of turnaround as a linear vs. non-linear process and the extent to which they considered the interactions between internal and external strategies to guide turnaround processes. As a result of the findings from this phase, and the consideration of success and failure in a dialectical relationship, we decided to develop a phase two that explored the experiences of both low-performing and high-performing organisations.

###  Phase 2 Results

 The initial search yielded published articles 3607 (see study selection procedure in Supplementary file 3). These were screened based on the title and type of article, resulting in 1386 articles. These articles were further screened on the basis of their abstracts, which left articles 111 for full-text review. Full-text review of these articles led to 41 articles that met the inclusion criteria. One additional article was identified by reviewing the bibliography, ultimately leading to 42 articles included in the review. We excluded articles that focused on improvement in individual pupil outcomes (ie, reading levels) and not general school performance. The reason for this exclusion was that these individual-level outcomes did not function as a determinant of organisational success or failure.

####  Characteristics of the Included Studies

 Seventeen of the studies took place in the United States, 20 were from the United Kingdom, 1 in the United Kingdom and the United States, 1 in Canada, 2 in Israel and 1 was a comparison across 6 European countries (see Table 1). The publications were relatively recent, with most articles published post-2010. Study designs varied, but most studies were qualitative, followed by quantitative and mixed-methods designs. Most of the articles were of average quality (as defined by the MMAT) with common limitations including low response rates in quantitative studies and lack of reflexivity in qualitative studies. Inter-rater reliability assessment indicated substantial agreement, with a kappa statistic of 0.80. For a full list of the included articles and their characteristics (including quality assessment), refer to Supplementary file 4.

####  Definitions of Failure and Turnaround

 We examined definitions of failure and success in the articles in relation to the thematic framework we developed in phase 1 (Supplementary file 2). Failure/success appeared to be defined in most studies as: “organisational performance that is persistently below or above some minimally acceptable level” (A3 in Supplementary file 2). Low performing and high performing organisations were defined as such in relation to nationally established ratings or indices (ie, Audit Commission Comprehensive Performance Assessment ratings or Academic Performance Index). This definition distinguished between the minimum acceptable level of performance, performance that is ‘persistently’ below and above this acceptable level. The focus of this definition of failure/success tended to be on the organisation and was not applied to wider system.

 Some studies have tried to incorporate a “success/failure as a system property” approach by considering the relationships between the provider organisation and other external organisations, but, even in these studies, consideration of system-level properties was limited. In most studies, failure was considered as produced by limited or dysfunctional organisational learning (B5 in Supplementary file 2). Preventing failure and producing improvements were dependent on changes in organisational culture. Some studies indicated that individual interventions aimed at quality improvement were not effective if they did not address problems in organisational culture.

 In relation to turnaround strategies, we found variation regarding if these were internally or externally driven. For instance, Jas and Skelcher^17^ argued that in order for turnaround to be effective in local authorities, it needed to be externally driven. In healthcare, most of the turnaround strategies were based on relational/mutual arrangements. Some studies framed turnaround under RRR (replacement, retrenchment and renewal) we previously identified in phase 1 (including these three aspects of the intervention or only some of them). Replacement can refer to the replacement of executive members of a Board, retrenchment is based on using stricter financial controls and focusing on performance targets and renewal strategies could involve changing organisational culture and improving stakeholder engagement.^3^ Most of the interventions we analysed followed a renewal approach (with few examples of replacement). In local authorities, retrenchment (that is, reduction of spending in particular areas) was seen as producing negative consequences.

####  Type of Intervention

 One of the aims of the review was to explore the types of interventions used to improve quality in low-performing and high-performing organisations. The types of interventions varied by sector, but we found overlap in a few of these (see Table 2). We were able to group the interventions in ten main categories: (1) Financial incentives (including pay for performance schemes, grants), (2) External partnerships and sharing of practice, (3) QI training, (4) Reorganisation at multiple levels, including senior leadership level and the use of external interim managers, (5) Development of existing leadership and/or middle management, (6) Identification of organisational goals or priorities, (7) Use of routine data and establishment of performance standards (including dashboards), (8) Standardising care practices, (9) 3 Rs, and (10) Interventions involving external inspections.

**Table 2:** Articles by Type of Intervention

**Intervention Type**	Education*	Local Government*	Healthcare*
Financial incentives (including pay for performance schemes, grants)	Rice et al^18^; Rosenberg et al^19^		Werner et al^20^
External partnerships and sharing of practice**	Marsh et al^21^		Mannion et al^22^
QI training and protected time for implementation of changes**			Hochman et al^23^
Reorganisation at multiple levels, including senior leadership level and the use of external interim managers	Heck and Chang^24^	Beeri and Navot^25,26^; Yapp^27^	Mannion et al^22^; Hochman et al^23^
Development of existing leadership and/or middle management	Meyers and Hitt^28^; Nicolaidou and Ainscow^29^; Orr et al^30^; Van Gronigen and Meyers^31^	Beeri^32^; Jas^33^	Gagliardi and Nathens^34^
Identification of organisational goals or priorities**	Finnigan et al^35^; Chapman and Harris^36^		Tsai et al^37^; Hochman et al^23^
Use of routine data and establishment of performance standards (including dashboards)**	Mintrop and Trujillo^38^	Turner et al^39^	Chang et al^40^; Rose^41^; Tsai et al^37^; Gagliardi and Nathens^34^; Aboumatar et al^42^
Staff engagement in the development of interventions**			Curry et al^7^
3 Rs		Beeri^26,43^	
Interventions involving external inspections	Willis^44^; Wilmott^45^; Parsons^46^; Perryman^47,48^; Gorton et al^49^; Ehren et al^50^	Jas and Skelcher^17^	Allen et al^51^; Boyd et al^52^; Castro-Avila et al^53^

* Some articles might present findings from the same study. ** Interventions identified as successful (see Table 3 for additional details).

####  Features of “Successful Interventions”

 Some of the articles described interventions that produced and maintained improvements in quality. The authors reflected on the features that made these interventions successful. These included the need to: (1) establish protected time for staff to implement the changes, (2) ensure staff engagement in the identification of problems and development of the interventions (to guarantee ownership), (3) develop strong relationships with other organisations (to share good practice), (4) identify clear goals and targets to meet as a result of the intervention and use data to monitor progress (see Table 3).

**Table 3:** Features of Successful Interventions

**Features**	**Examples**	**Articles**
Protected time for staff to implement the changes	Maintaining adequate staffing levels to ensure staff can implement changes Allow staff to maintain protected time for QI	Hochman et al^23^
Staff engagement in the identification of problems and development of the interventions	Engaging staff across all departments	Chang et al^40^; Curry et al^7^
Develop strong relationships with other organisations	Sharing good practice with other organisations Peer to peer learning Supportive partnerships	Mannion et al^22^; Aboumatar et al^42^; Brewster et al^8^; Marsh et al^21^; Jas et al^17^; Gorton et al^49^
Identify clear goals and targets to meet as a result of the intervention and use data to monitor progress	Pro-active use of data Routine review of quality metrics Dashboards with performance metrics Use of patient experience data	Brewster et al^8^; Gagliardi et al^34^; Hochman et al^23^; Mintrop et al^38^

####  Issues to Consider in Implementation

 Our review confirmed the findings of previous reviews that have stated that improvement interventions are shaped by the organisational culture, where negative cultures were framed by limited ownership, lack of collaboration, hierarchies and disconnected leadership.^6^ Successful improvement interventions were implemented in organisations where the culture was characterised by staff with a ‘can do attitude’, the desire to improve, and engaged leadership (ie, empowering staff and involving them in decision-making processes). There were also reflections on the need to consider processes of implementation. For instance, in the case of school inspections, Ehren et al^50^ argued that while these might be beneficial for schools, this was contingent on the content of the feedback and how the feedback was communicated to schools after inspections. The authors found that feedback that included detailed information on performance expectations and a clear understanding of current teaching conditions was more effective.^50^ Studies on the process of carrying out inspections in healthcare have indicated that inspections were more reliable if carried out by larger teams, if inspectors were allowed to have discussions and received appropriate training.^52^

####  No Effects, Potential Negative Outcomes and Unintended Consequences

 Some of the studies in schools highlighted the negative consequences of being labelled as a failing organisation, for instance, there were important implications for recruitment and retention of both staff and pupils, relationships with parents and the community and links with the local authority. Chapman and Harris^36^ found that top-down reform that treats all schools as the same is unlikely to secure long-term improvement and change as they should be free to select the approaches to change that suit their particular needs. External pressures were also seen as negative as in some cases, they resulted in the “repackaging” or “recycling” of ideas and approaches (in the case of this study, restructuring plans) that did not support the meeting of organisational goals or contribute to learning.^35^ The external vs. internal debate was also present in studies focusing on school inspections and special measures, where some authors argued in favour of the use of school self-evaluations rather than external inspections.^49^ Recent research on the use of inspections in healthcare has also shown no effects in performance generated by external inspections.^51,53^

####  Costs Associated With the Interventions

 We found that limited attention was paid to the costs of the interventions or cost-savings produced by the interventions. Furthermore, the studies did not explore opportunity costs involved in implementing the interventions, the use of the time and resources to make different changes and the extent to which the changes could have been carried out without the intervention.

####  Implications for Future Research 

 The articles included in the review identified gaps in research. One proposal was to explore how turnaround strategies change through time, taking into consideration the historical context of organisations. Many studies only captured a snapshot of the intervention and organisational culture, missing the nuances of how change was negotiated. Another gap identified was the need to take into account whether improvement approaches were internally- or externally-driven interventions by regulatory bodies for example. The role of external partnerships in creating and sustaining improvements is currently being explored in an ongoing review,^54^ yet additional work is required to identify the components of interventions that might respond better to internal drive, versus those that might benefit from external support (or a combination of both). In relation to this, some articles highlighted the need to make sure that studies of these types of intervention capture the experiences of staff members across all layers of the organisation, particularly front-line staff and lower management (as many studies have focused on changes taking place at senior leadership levels).

## Discussion

**Figure 2:**
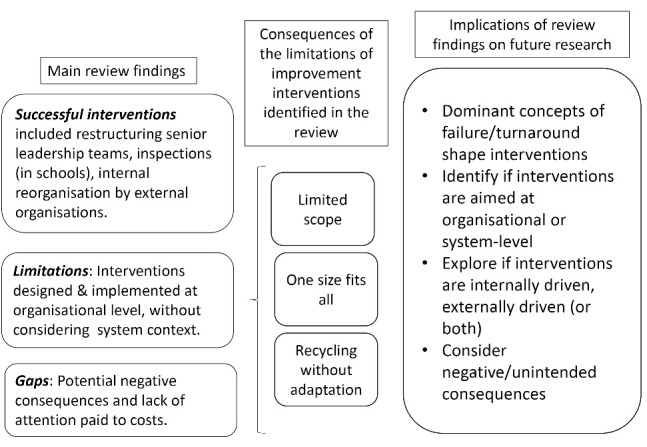
Summary of Main Findings and Implications for Future Research.

 In this review, we explored the delivery of improvement interventions for low and high performing organisations while considering the underlying concepts used to define success/failure and turnaround (see summary of findings and implications in Figure 2). We found that most improvement strategies in health, education and local government settings continue to define failure in relation to the inability of organisations to meet pre-established performance standards. Turnaround is often considered as a linear process designed to fix problems and bring organisations up to the ‘appropriate’ level. In most cases, the causes of failure and success are considered in relation to organisational features or characteristics (ie, organisational culture, leadership arrangements), without a wider consideration of the system where these organisations operate or their history. Improvement interventions are designed accordingly, that is, are focused on specific areas of the organisation or the organisation as a whole, with limited system-level thinking.

 The literature we reviewed has pointed to the problems associated with the definitions outlined above. Some authors have highlighted the limited scope of some interventions which did not take into consideration issues at a system level, such as regional financial pressures, fragmented care and workforce challenges eg, recruitment and retention in particular geographical areas. Others have also questioned the rollout of “one size fits all” interventions across multiple organisations, without recognising the need to adapt improvement interventions to the local context. The recycling of ideas from other organisations that did not suit the local context was also found in some studies.

 Related to the last point, a domain of literature that did not come up explicitly in this review (because of the key words and targeted focus), yet has relevance, is the process of recycling popular ideas to support quality improvement and performance that originate from external organisations, sectors and global institutions. This is a theme long explored by institutional theorists who trace the flow and adoption of different types of knowledge found internationally.^55-57^ This review identified some studies where the re-use of ideas was not carried out by taking into consideration the local context.^35^ Our findings are also supported by the knowledge mobilisation literature, which suggests that whilst ideas for improvement may easily spread across boundaries, they might not achieve local buy-in and a good ‘epistemic fit’ within local contexts, especially if there is a lack of knowledge brokering and senior support to encourage organisations to be receptive to the new ideas.^58^

 The findings in relation to the implementation of successful interventions mirrors other analyses of improvement interventions, where success is often associated with staff engagement, protected staff time for implementation, clear priority-setting and the use of routine data to monitor progress at Board level.^37,59-61^ An interesting finding was a list of potentially negative and unintended consequences of implementing interventions, particularly for low-performing organisations. Partially, this negative effect had to do with the labelling of organisations as low-performing and requiring interventions. This labelling, for instance, by placing organisations in “recovery” or “special measures” programmes, negatively impacted on staff morale, retention and recruitment. It also meant organisations became under additional scrutiny.

 We were surprised by the limited consideration of the costs of designing and delivering improvement interventions, especially as many low-performing organisations appeared to be suffering from financial difficulties. In addition to a more in-depth consideration of the impact of these interventions on costs and potential cost savings, the literature we reviewed pointed to the need to develop additional research on the changes in turnaround strategies through time and the interaction between internally-driven improvements and external processes.

 Limitations include articles missed: although we employed multiple broad search terms and a phased search strategy, it is possible that we missed articles that did not use these terms. The broad scope of the review on health, education and local government could mean that some aspects of organisational performance might not be comparable and the breadth of the review might have limited more in-depth analyses. We used self-identified definitions of low and high performing organisations instead of external validated metrics and thresholds. The tool we used to assess the quality of the studies, the MMAT, also has limitations that have been discussed elsewhere.^15^

## Conclusions

 There is a limited understanding on the effectiveness of improvement interventions in supporting failing organisations and improving the quality of care in high-performing organisations. The aim of this review was to examine the underlying concepts guiding the design of these interventions, processes of implementation, the unintended consequences of implementing the interventions, and their impact on costs and quality of care.

 We found dominant definitions of success/failure and turnaround, which have impacted on the design and implementation of improvement interventions. The limitations of these definitions have been the reduced scope of the interventions, the linear perception of turnaround, and lack of consideration of organisations within the wider system in which they operate. Future research should focus on identifying the dominant concepts of failure/turnaround shaping interventions implemented in low-performing organisations; determine if interventions are aimed at organisational or system-level or are internally or externally driven interventions; document the costs of delivering the interventions and explore any negative and/or unintended consequences of implementing interventions aimed at the improvement of organisational performance.

## Acknowledgements

 We thank Angus Ramsay, Christine Taylor, Chris Sherlaw-Johnson, Melissa Hill, Sonila Tomini, and Pei Li Ng (Department of Applied Health Research, UCL) for critical review of the manuscript and administrative support of the study. We would also like to thank Kieran Walshe and Gill Harvey for their feedback on phase 1 of the review. This manuscript is independent research funded by the NIHR (Health Services and Delivery Research, 16/138/17 – Rapid Service Evaluation Research Team). NJF is an NIHR Senior Investigator. NJF and ECB were supported in part by the NIHR Collaboration for Leadership in Applied Health Research Care (CLAHRC) North Thames at Barts Health NHS Trust.

## Ethical issues

 Not applicable.

## Competing interests

 Authors declare that they have no competing interests.

## Authors’ contributions

 NJF, CVP, ECB, and JL contributed to the conception and design of the review. CVP led on the drafting of the manuscript. All authors contributed to revision of the manuscript and approved the final manuscript.

## Funding

 This manuscript is based on independent research funded by the NIHR (Health Services and Delivery Research, 16/138/17 – Rapid Service Evaluation Research Team).

## Disclaimer

 The views expressed are those of the authors and not necessarily those of the NHS, the NIHR or the Department of Health and Social Care.

## Supplementary files


Supplementary file 1. Search Strategy Design Using PICOS.

Supplementary file 2. Thematic Framework on Organisational Failure and Turnaround Based on Review of Theoretical Content.

Supplementary file 3. Study Selection Procedure.

Supplementary file 4. Characteristics of Studies Included in Phase 2 and Quality Assessment Results.
