# Supplementary material for: The Implementation of Improvement Interventions for "Low Performing" and "High Performing" Organisations in Health, Education and Local Government: A Phased Literature Review
Source: Int J Health Policy Manag. 2020 Nov 1;11(7):874–82. doi: 10.34172/ijhpm.2020.197 (PMC9808185; doi:10.34172/ijhpm.2020.197)
Supplement: Supplementary file 1 — Search Strategy Design Using PICOS. [file ijhpm-11-874-s001.pdf]

Supplementary file 1. Search Strategy Design Using PICOS

**Population**	High-performing and low-performing organisations in healthcare, education and local government settings
**Intervention**	Interventions, support, quality improvement projects designed to improve healthcare quality, organizational changes, changes in leadership
**Comparison**	None
**Outcome**	Quality improvement
**Setting**	Healthcare, education, local government

Search strategy example

1. Failing OR low-performing OR struggling OR challenged OR "special measures" OR turnaround OR "high-performing" OR successful

AND

Hospitals OR healthcare organisation OR healthcare facilities OR healthcare administration OR provider

AND

Intervention OR support OR "quality improvement" OR improvement OR "Virginia Mason" OR "Lean method" OR "Lean thinking" OR "measuring for improvement" OR "Six Sigma" OR PDSA OR "improvement science" OR "statistical process control" OR "organizational change" OR "leadership change" OR "absorptive capacity" OR "replacement, retrenchment and renewal"

2. Failing OR low-performing OR struggling OR challenged OR "special measures" OR turnaround OR "high-performing" OR successful

AND

Education OR schools OR "educational setting"

AND

Intervention OR support OR “quality improvement” OR improvement OR “Virginia Mason” OR “Lean method” OR “Lean thinking” OR “measuring for improvement” OR “Six Sigma” OR PDSA OR “improvement science” OR “statistical process control” OR “organizational change” OR “leadership change” OR “absorptive capacity” OR “replacement, retrenchment and renewal”

3. Failing OR low-performing OR struggling OR challenged OR “special measures” OR turnaround OR “high-performing” OR successful

AND

“local government” OR “local authority” OR government

AND

Intervention OR support OR “quality improvement” OR improvement OR “Virginia Mason” OR “Lean method” OR “Lean thinking” OR “measuring for improvement” OR “Six Sigma” OR PDSA OR “improvement science” OR “statistical process control” OR “organizational change” OR “leadership change” OR “absorptive capacity” OR “replacement, retrenchment and renewal”
