# Supplementary material for: The Implementation of Improvement Interventions for "Low Performing" and "High Performing" Organisations in Health, Education and Local Government: A Phased Literature Review
Source: Int J Health Policy Manag. 2020 Nov 1;11(7):874–82. doi: 10.34172/ijhpm.2020.197 (PMC9808185; doi:10.34172/ijhpm.2020.197)
Supplement: Supplementary file 2 — Thematic Framework on Organisational Failure and Turnaround Based on Review of Theoretical Content. [file ijhpm-11-874-s002.pdf]

Supplementary file 2. Thematic Framework on Organisational Failure and Turnaround Based on Review of Theoretical Content

**Domain**	**Description**	**Disciplines [classified based on journal]**	**Examples in the literature**
**ORGANISATIONAL FAILURE/SUCCESS**			
A. Concepts used separately or in combination			
1. Failure as decline	\`Existence-threatening decline' in the performance of the organisation. Focus is on the organisation.	Management, business	Pandit 2000; Mellahi and Wilkinson 2004
2. Failure as crisis	Focuses on the peak of failure, considering it as an acute process or sudden onset (considers the possibility of crisis denial and hidden crisis to account for more gradual representations of failure). Focus is on the organisation.	Management, business	Slater 1984; Weitzel and Jonsson 1989
3. Failure as below acceptable performance levels	Organisational performance is persistently below some minimally acceptable level. Distinguishes between the minimum acceptable level of performance; and performance which is \`persistently' below this acceptable level. Focus is on the organisation.	Management, business	Hambrick 1983
4. Success as a system property/structural processes involved in failure	High performance results from properties of the system and not characteristics of the individuals. Focus is beyond the organisation and attention is paid to the structures of inspection and performance.	Health services research, education	Baker et al. 2008; Bate 2007; Taylor et al. 2015; Willmott 1999; Perryman 2005

B. Theoretical frameworks			
1. Industrial Organization (IO)	Organisational failure is caused primarily by changes in the external environment, which are the result of a range of technological, economic, regulatory and demographic factors.	Management, business	Mellahi and Wilkinson 2004
2. Organisational Ecology theory (OE)	Applies a natural selection model to organisational dynamics. It is based on a biological analogy, in which organisations scan the environment and compete and recognise situations, mechanisms and processes underlying emergence, growth, regulation and demise	Management, business	Hannan and Freeman 1989
3. Life Cycle (ILC) theory	Organisational failure is considered a natural and objective event (i.e. likely to occur), the outcome of factors such as saturation of demand, running out of supplies, and introduction of new technology.	Management, business	Klepper 1997
4. Organisational Psychology (OP)	Views failure and success as a result of internal rather than external and environmental factors (i.e. leadership capacity, composition of top management teams, governance models, organisational arrogance and myopia)	Management, business; health services research	Mellahi et al. 2002; Curry et al. 2011*
5. Failure and organisational learning/organisational culture, role of emotions	Failure is caused by lack, limited or dysfunctional organisational learning. Preventing failure is dependent on changes in organisational culture.	Health services research, education	McKiernan 2002; Fulop et al. 2005; Walshe and Shortell 2004; Harvey et al. 2015; Jones et al. 2017; Dixon-Woods et al. 2014; Willis 2010; Nicolaidou and

			Ainscow 2005; Leithwood et al. 2008
6. Failure and success within regimes of surveillance	Foucauldian outlook on surveillance, monitoring, and performance, seeing external actors such as Ofsted as reproducing disciplinary power. Critique of dualisms. Focus on discourse and view of inspections (or the threat of inspections) as the mechanism through which discipline is reproduced.	Education	Willmott 1999; Perryman 2005, 2006; McGivern et al. 2017; Ferlie et al. 2012
7. Contextual factors leading to failure (internal and external)	Failure is usually caused by a combination of different factors (both internal and external). Management is unlikely to be the sole cause of decline and managers may be symptoms of failure as much as causes. Recognises the need to consider system-wide factors when addressing failure.	Management, business, health services research, education	Fulop et al. 2005; Walshe et al. 2004; Ravaghi et al. 2015; Smithson et al. 2018; Taylor et al. 2015; Vaughn et al. 2018; Dixon-Woods et al. 2014; Chapman 2002, 2004;
**TURNAROUND**			
C. Concepts			
1. Turnaround as recovery	“The actions taken to bring about recovery in performance in a failing organisation".	Management, business	Pandit 2000
2. Turnaround as a potential stage in wider analysis of organisational failure	Explains the process of decline and/or turnaround as occurring in sequential stages that may result in the survival and	Management, business	Chowdhurry, 2002; Fulop et al. 2005;

(i.e. McKieran's six stage model, Argyowasmy's two stage model)	performance improvement or failure of an organisation.		Paton and Mordaunt 2004
3. Turnaround as a complex (non-linear) process	Considers turnaround as a complex process involving intra-organizational areas (including human relations, organizational behavior and group-level behavior). Considers turnaround in the context of any radical organizational change and not just recovery from a crisis.	Management	Beeri 2009, 2012
D. Theoretical exploration of turnaround strategies			
1. Turnaround based on replacement	Strategies put in place to reshape organisational structures.	Management, business, Health services research	Harvey 2005a; Fulop et al. 2004; Paton 2004; Walshe et al., 2004; Jas and Skelcher, 2005; Ravaghi 2007
2. Turnaround based on retrenchment	Strategies are put in place to limit the use of resources and "save" the organisation (i.e. cost cutting, asset reduction).	Management, business, Health services research	McKiernan, 2003; Greenhalgh, 1983;<br><br>Hardy, 1987; Sutton et al., 1986; Ravaghi 2007
3. Turnaround based on renewal	Activities utilised to reorient the direction of an organisation and its vision, with the aim of ensuring long-term successful survival.	Management, business, Health services research	Protopsaltis et al. 2002; Fulop et al. 2004; Walshe et al., 2004; Boyne, 2008; Ravaghi 2007

4. Turnaround based on external episodic intervention	Hands off and focused on performance outcomes and not process. Use of regular inspections.	Management, business, Health services research	Jas and Skelcher 2004; Harvey 2006
5. Turnaround based on relational/mutual arrangements	There is agreement between all parties and it is based on monitoring.	Management, business, Health services research	Jas and Skelcher 2004; Harvey 2006
6. Turnaround based on mandated approaches	External actors take over the organisation, allowing little room for negotiation.	Management, business, Health services research	Jas and Skelcher 2004; Harvey 2006

*We have associated this article with the organisational psychology framework because it is grounded in the positive deviance concept (this was our association and not the association of the authors).
