# Supplementary figures and images for: The Implementation of Improvement Interventions for "Low Performing" and "High Performing" Organisations in Health, Education and Local Government: A Phased Literature Review
Source: Int J Health Policy Manag. 2020 Nov 1;11(7):874–82. doi: 10.34172/ijhpm.2020.197 (PMC9808185; doi:10.34172/ijhpm.2020.197)

### Supplementary file 3. Study Selection Procedure

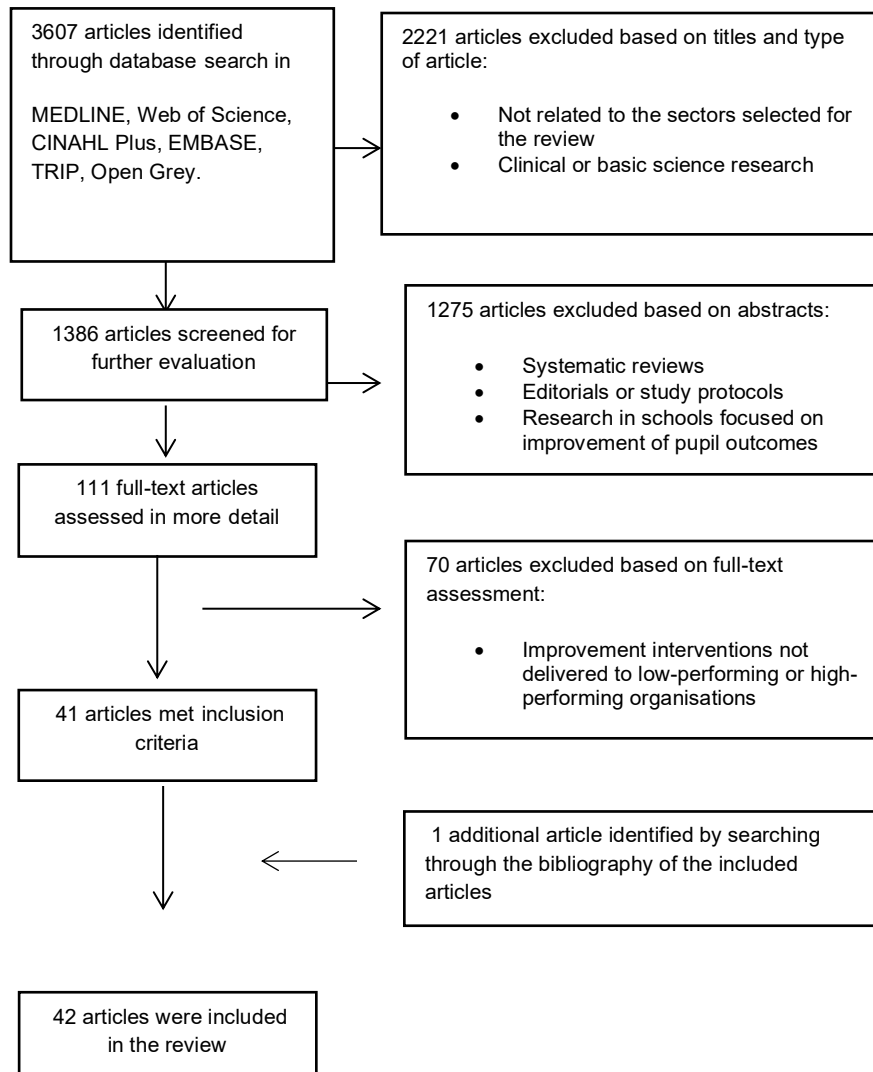

Supplement: Supplementary file 3 — Study Selection Procedure. [file ijhpm-11-874-s003.pdf]
