# Supplementary material for: The Implementation of Improvement Interventions for "Low Performing" and "High Performing" Organisations in Health, Education and Local Government: A Phased Literature Review
Source: Int J Health Policy Manag. 2020 Nov 1;11(7):874–82. doi: 10.34172/ijhpm.2020.197 (PMC9808185; doi:10.34172/ijhpm.2020.197)
Supplement: Supplementary file 4 — Characteristics of Studies Included in Phase 2 and Quality Assessment Results. [file ijhpm-11-874-s004.pdf]

Supplementary file 4. Characteristics of Studies Included in Phase 2 and Quality Assessment Results

First author surname	Year of publication	Country where the study took place	Sector where the study took place	Study design	Quality assessment *
Beeri	2009	UK	LA	Qualitative	***
Beeri	2012	UK	LA	Mixed-methods	***
Beeri	2013a, 2013b	Israel	LA	Mixed-methods	***
Beeri	2014	UK	LA	Quantitativ e	**
Chapman	2002	UK	Schools	Qualitative	***
Chapman	2004	UK	Schools	Qualitative	***
Ehren	2013	6 European countries	Schools	Qualitative	***
Finnigan	2012	USA	schools	Mixed-methods	**
Gorton	2014	UK	schools	Qualitative	***
Heck	2017	USA	schools	Quantitativ e	***
Rosenberg	2015	USA	schools	Mixed-methods	***
Jas	2005	UK	LA	Qualitative	***
Marsh	2017	USA	schools	Qualitative	***

Meyers	2018	USA	schools	Mixed-methods	***
Nicolaidou	2005	UK	schools	Qualitative	***
Perryman	2005, 2006	UK	schools	Qualitative	***
Mintrop	2007	USA	Schools	Quantitative	**
Parsons	2013	UK	schools	Quantitative	***
Rice	2012	USA	schools	Qualitative	***
Orr	2008	USA	schools	Qualitative	***
Turner	2004	UK	LA	Mixed-methods	**
VanGronigen	2019	USA	schools	Qualitative	***
Willis	2010	UK	Schools	Qualitative	***
Wilmott	1999	UK	Schools	Qualitative	***
Yapp	2007	UK	LA	Qualitative	***
Chang	2018	US	healthcare	Qualitative	***
Rose	2012	US	Healthcare	Qualitative	***
Curry	2011	US	Healthcare	Qualitative	***
Mannion	2005	UK	Healthcare	Qualitative	***
Werner	2008	US	Healthcare	Quantitative	***

Tsai	2015	US and UK	Healthcare	Quantitative	***
Hochman	2016	US	Healthcare	Qualitative	***
Gagliardi	2015	Canada	Healthcare	Qualitative	***
Aboumatar	2015	US	Healthcare	Quantitative	***
Brewster	2011	US	Healthcare	Qualitative	***
Mills	2008	US	Healthcare	Qualitative	***
Freed	2005	US	Healthcare	Qualitative	***
Allen	2019	UK	Healthcare	Quantitative	***
Boyd	2016	UK	Healthcare	Mixed-methods	***
Castro-Avila	2019	UK	Healthcare	Quantitative	***

MMAT quality assessment categories range from * (lowest) to **** (highest).
